# Commentary: Editorial: The Psychological and Physiological Benefits of the Arts

**DOI:** 10.3389/fpsyg.2022.869038

**Published:** 2022-03-22

**Authors:** Christopher Bailey

**Affiliations:** Arts and Health Lead Department, World Health Organization, Geneva, Switzerland

**Keywords:** art, health, creative arts therapies, arts and health research, cultural policy, health policy, creativity

The 1947 Constitution of the World Health Organization states, “Health is a state of complete physical, mental and social wellbeing and not merely the absence of disease or infirmity.” (World Health Organization, 2020). With this in mind, WHO has always used the arts in its work on health promotion and communication. However, this effort was given serious focus with the publication of the first scoping review on the evidence for the health benefits of the arts (Fancourt and Finn, 2019) published by the WHO Regional Office for Europe. With the publication of this report, we have affirmed what so many in the diverse field of arts and health have long known: *the arts are a vital partner in our health, care, and wellbeing*.

What remains now is to advance our understanding on specifically when, how, and with whom the arts contribute to positive psychological, physiological, and social wellbeing. We need to drill down to the evidence base of the health benefits of the arts and to areas that have stood out demographically. One area that is attracting attention is the older generation: there is a demographic boom and associated expansion of conditions, diseases and challenges of growing older. An anecdote from my own experiences: my father in law suffers from dementia, and he is in a constant state of emotional anxiety. When we ring the doorbell, he answers the phone. When we try to talk to him, he is hearing voices of people long dead around him. And yet when he plays the piano, he experiences these moments of concentration of clarity and of peace, that are really quite remarkable. The second important area is younger people, like my son, who are also going through great anxiety right now. They are losing faith in the older generation to make the right decisions to make this planet habitable for the next generation. They are losing faith in the ability of the community to share and collaborate and care for one another. Through the arts, in his case film, my son is trying to create a narrative that rediscovers that connection between people to find a way forward. And of course it is also all of us, going through the third year of a pandemic that has kept us apart, resulted in losses of people, property and ways of living and challenged our mental health. For me, the healing power of the arts lies in the hope it has brought to me and to so many during this time.

This Research Topic on the psychological and physiological benefits of the arts is a significant contribution to this evidence base. The 82 articles offer insights into the benefits of participating in the arts for the general population as well as treatment outcomes for specific populations such as the role of dance and dance movement therapy in alleviating depression and improving gait for people with Parkinson's disease; the use of art and art therapy to support children and adults in working through and communicating mental health concerns; the role of theater, drama therapy, and psychodrama to increase social connection amongst neurodiverse youth, and reduce stigma and symptoms of PTSD; and the use of music and music therapy in pain reduction and quality of life in patients with cancer. This collection also offers a rigorous analysis of therapeutic change factors involved in the creative arts therapies which gives us insight into why and how these clinical practices facilitate change.

We have much more to discover about *how* the arts can change how we feel, think, and behave. Given the collective challenges we face now from social inequity, the pandemic, climate change, and sustainable peace, we need to invest in artists, arts therapists, and in alliances between the arts and wellbeing at all levels of government, education, and care provision. Research, like the kind demonstrated in this excellent collection of articles, is an essential part of this investment and offers important avenues for policy. Working with UNESCO, the WHO can imagine a day in the not too distant future where both organizations jointly present evidence with relevant policy recommendations to a combined convening of ministers of health and culture, ushering in a new era of investment in the arts, culture, and creativity, not simply for its own sake or the creative economy, but as an investment in the health of communities.

## Author's Note

Christopher Bailey is the Arts and Health lead at the World Health Organization, based in Geneva, Switzerland. His program focuses on the research agenda, community implementation and mobilizing the global media to explore, understand and support the health benefits of the arts, in everyday life as well as an instrument in the field. Educated at Columbia and Oxford Universities, as well at the American Academy of Dramatic Arts, before entering Global Health and Philanthropy, Bailey was a professional actor and playwright. He served as Research Manager of the Rockefeller Foundation before joining the World Health Organization as their Knowledge Management Advisor. At WHO he has also led the Knowledge Communities and Strategies group working in Health Informatics particularly in rural Africa, and later was the Coordinator of Online Communications.

## Author Contributions

The author confirms being the sole contributor of this work and has approved it for publication.

## Conflict of Interest

The author declares that the research was conducted in the absence of any commercial or financial relationships that could be construed as a potential conflict of interest.

## Publisher's Note

All claims expressed in this article are solely those of the authors and do not necessarily represent those of their affiliated organizations, or those of the publisher, the editors and the reviewers. Any product that may be evaluated in this article, or claim that may be made by its manufacturer, is not guaranteed or endorsed by the publisher.
